# Economic burden of acute coronary syndrome in South Korea: a national survey

**DOI:** 10.1186/1471-2261-13-55

**Published:** 2013-08-08

**Authors:** Jinhyun Kim, Eunhee Lee, Taejin Lee, Aeree Sohn

**Affiliations:** 1 College of Nursing/The Research Institute of Nursing Science, Seoul National University, 28 Yeongeon-dong, Jongro-gu, Seoul, Republic of Korea; 2 Graduate School of Public Health, Seoul National University, Seoul, Korea; 3 Department of Health Management, Sahmyook University, Seoul, Korea

**Keywords:** Acute coronary syndrome, Cost of disease, Unstable angina, Myocardial infarction, Ischemic heart disease

## Abstract

**Background:**

Acute coronary syndrome (ACS) is highly prevalent in Korea and is the third-leading cause of death in Korea; however, the economic cost of ACS on Korean society has not been investigated. This study examined the economic effect of ACS on the Korean population during the period 2004 to 2009.

**Methods:**

The analysis used the cost of illness (COI) framework. Data on direct medical costs, direct non-medical costs, and productivity loss related to ACS morbidity and mortality were included. The Korean National Health Insurance Corporation’s claim database was used to obtain data on annual healthcare utilization and expenditures for the entire South Korean population. By using a data mining technique, we identified healthcare claims with ACS-related disease codes. Costs were estimated by using a macro-costing method.

**Results:**

In 2009, the prevalence of ACS in Korea was 6.4 persons per 1,000 population members and the associated mortality rate was 20.2 persons per 100,000 population members. The total cost of ACS in 2009 was USD 918.2 million. Of the total, direct medical cost was USD 425.3 million, direct non-medical cost was USD 11.4 million, and cost associated with morbidity and mortality was USD 481.5 million.

**Conclusions:**

The results show that the total cost of ACS to the Korean society is high. Early and effective management of ACS is required to reduce ACS-associated mortality and morbidity. We suggest that further research be undertaken to determine ways to reduce the economic effects of ACS and its treatment.

## Background

Acute coronary syndrome (ACS), which includes unstable angina (UA), non-ST-segment elevation myocardial infarction (MI) and ST-segment elevation MI, is highly prevalent in Korea and is the third-leading cause of death in South Korea [1]. Moreover, ACS is the most common condition related to ischemic heart disease in the West, and it is the major condition associated with heart disease requiring hospitalization [2-4]. According to the Korean National Statistical Office, the current level of morbidity and mortality associated with ACS has increased markedly from the level of 10 years ago [1]. In a previous study, the treatment cost associated with cardiovascular disease in Korea was USD 1.38 billion in 2001 and USD 2.25 billion in 2005 while the COI was USD 5.13 billion in 2001 and USD 5.89 billion in 2005 [5]. Since the aging index in Korea is estimated to increase from 67.7% in 2010 to 213.8% in 2030 [1], cardiovascular disease is expected to increase steadily for metabolic syndrome according to aging [6]. Thus, the prevalence of ACS and its associated costs are also expected to increase markedly. However, the economic effect of ACS on the Korean society has not been previously reported. In this study, we report on the economic burden of ACS in Korea over between 2004 and 2009.

## Methods

### Study design

**Figure 1:**
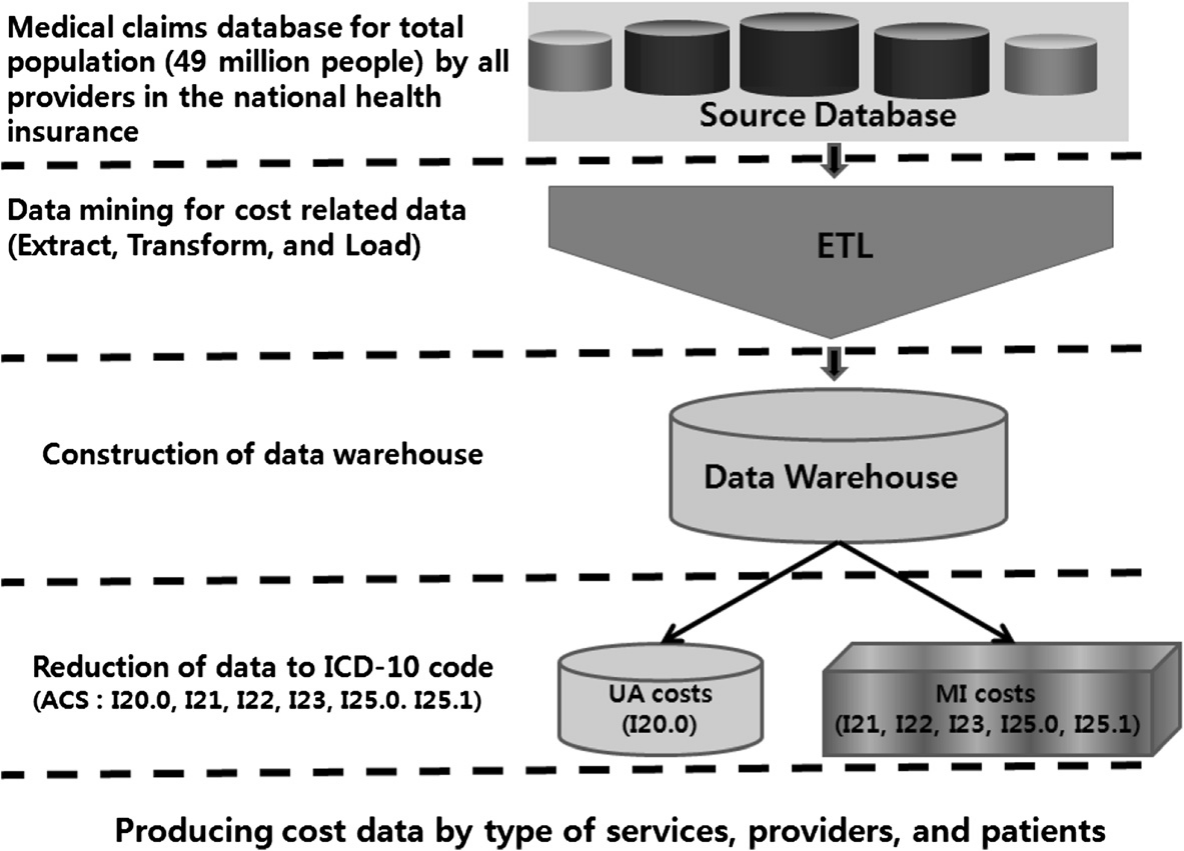
Process of extracting ACS data from the national health insurance database.

This retrospective study analyzed data from the Korean National Health Insurance Corporation’s (NHI) claims database that included medical claim information for the entire South Korean population (approximately 49 million people). The database includes information on health care expenditures as well as financial and reimbursement status. This large, longitudinal database provides integrated enrollment, medical, and prescription data for the entire Korean population, which can be stratified by age and gender (Figure 1).

Institutional review board and ethic committee approvals were not required for this study.

### Patient identification

The study population included patients with UA or MI who were discharged from hospitals from 2004 to 2009. Patients enrolled included those with medical claims with the International Statistical Classification of Diseases and Related Health Problems 10th Revision (ICD-10) codes for ACS and included I20.0 (UA), I21 (acute MI), I22 (subsequent MI), I23 (certain current complication following acute MI), I25.0 (atherosclerotic cardiovascular disease, sp described), and I25.1 (atherosclerotic heart disease) of MI. Codes I25.0 and I25.1 are chronic diseases and examination and treatments such as creatine kinase muscle-brain (CK-MB), troponin, coronary artery bypass graft (CABG) and percutaneous coronary intervention (PCI) for MI patients are frequently claimed with those codes at several medical institutions in Korea; thus, the positive rate of MI associated with those codes was high in a previous study [7]. To avoid underestimation of MI prevalence and to allow comparison with previous results, our study included codes I25.0 and I25.1.

### Cost

The analysis used a COI framework and included direct medical costs, direct non-medical costs, and indirect costs such as productivity loss from ACS-related morbidity and mortality [8,9]. Our estimate of total societal cost (SC), which included direct costs (DC) and indirect costs (IDC), was developed by using a macro-costing method.

The DC incurred when treating ACS included direct medical costs (MC), such as hospitalization and outpatient costs, and direct non-medical costs (NMC), such as transportation and caregiver costs.

The main payment system for healthcare in Korea is based upon a fee-for-service (FFS). All MC are determined within that FFS system. We used the NHI claims database to determine NHI payments and a national survey report [10,11] to determine out-of-pocket (OOP) payments. The ACS-related NHI payment was obtained by summing payments for each ICD code extracted from the NHI database. To determine OOP payments, we applied a previously reported ratio of OOP payment to NHI payment for ACS [11]. Total MC included NHI hospitalization and outpatient expenditures and NHI-related OOP expenditures:

(1) $$\mathrm{MC}=\sum_{i\;j}\sum \left(\mathrm{InNH}I_{\mathrm{ij}}+\mathrm{OutNH}I_{\mathrm{ij}}\right)+\mathrm{OO}P_{\mathrm{ij}},$$

InNHI_*ij*_ = NHI payment for hospitalization expenditures, OutNHI_*ij*_ = NHI payment for outpatient expenditures, OOP = out-of-pocket payment, *i* = 0,1,…, n (age), and *j* = 1 or 2 (gender).

The NMC included transportation expenditures when patients visited a medical center and caregiver expenditures during hospitalization. Since a variety of transportation types were used by patients, we estimated transportation cost by using the average transportation costs reported in a 2007 Korean national health and nutrition survey [12], adjusted by the 2004 and 2009 changes in the Korean transportation price index. In our estimation of caregiver costs, we excluded costs related to informal care provided by relatives because of insufficient data on the characteristics of the relatives. Caregiver cost was calculated by using the average caregiver costs reported in the 2007 Korean national health and nutrition survey [12], adjusted by the other-medical-service price indices for 2004 and 2009.

(2) $$\mathrm{NMC}=\sum_{i\;j}\sum \left\{\left(\mathrm{In}O_{\mathrm{ij}}+\mathrm{Out}O_{\mathrm{ij}}\right)\times M\right\}+\sum \sum \left(\mathrm{In}N_{\mathrm{ij}}\times I\right)\;$$

InO_*ij*_ = number of hospitalization visits, OutO_*ij*_ = number of outpatient visits, M = transportation costs (based on round-trip transport), InN_*ij*_ = length of hospitalization, I = caregiver costs per day, *i* = 0,1,…,n (age), and *j* = 1 or 2 (gender).

The Human Capital Approach [13] was used to estimate the IDC of lost wages (LW) and lost future income (LFI) due to morbidity. To estimate the productivity loss due to morbidity, non-productivity days were calculated by summing hospitalization days and one-third of the number of outpatient days, as previously described [5]. Since outpatient-related productivity loss is less than hospitalization-related loss, we reduced outpatient days by two-thirds of the non-productivity rate reported previously [5]. After determining the non-productivity days, we estimated the productivity loss due to morbidity by multiplying the non-productivity days by the labor force participation rate, the employment rate, and by the average daily earnings for each age and gender [14].

(3) $$\begin{array}{l} \mathrm{LW}=\sum \sum_{i\;j}\left\{\left(\mathrm{In}N_{\mathrm{ij}}\times p_{\mathrm{ij}}\times e_{\mathrm{ij}}\times y_{\mathrm{ij}}\right)\right)+\\ \;\left(\left(\mathrm{Out}N_{\mathrm{ij}}\times 1/3\times p_{\mathrm{ij}}\times e_{\mathrm{ij}}\times y_{\mathrm{ij}}\right)\right\} \end{array}$$

InN_*ij*_ = length of hospitalization, p_*ij*_ = labor force participation rate, e_*ij*_ = employment rate, y_*ij*_ = average daily earning, OutN_*ij*_ = outpatient visiting days, *i* = 0,1,…,n (age), and *j* = 1 or 2 (gender).

The lost income due to illness (LFI) was estimated from death-related statistical data in the Korean National Statistics Office database [1]. The calculation of LFI converted future income of the deceased patient into a present value. To estimate LFI, we assumed no production activity for patients aged under 15 years and over 65 years. Data on patients that died due to ACS were used, by multiplying the period, average earnings, labor force participation rate, and employment rate according to age and gender (equation 4), to calculate the future period of production activity and to estimate lost income. Based on a previous study, the discount rate (r) for converting future income to present value was 0% [15]. Future income was increased by the previously reported wage growth rate. However, the average wage growth rate for the past 4–5 years ranged between 4.8% and 5.4%, which was similar to the reported 5% discount rate in the health care sector; therefore, we applied a discount rate r of zero. Equation 4 presents the calculation method used to determine LFI

(4) $$\mathrm{LFI}=\sum \sum_{i\;j}\left\{F_{\mathrm{ij}}\times \left(Y_{j}^{{{}^{t}}^{+\tau }}\times p_{\mathrm{ij}}\times e_{\mathrm{ij}}\right)/{\left(1+r\right)}^{{}^{t}}\right\}\;$$

F_*ij*_ = number of deaths, Y_*j*_^*t*+τ^ = average expected income in year *t* + τ (*t* = age at death and τ = life year), p_*ij*_ = labor force participation rate, e_*ij* =_ employment rate, r = discount rate, *i* = 0,1,…,n (age), and *j* = 1 or 2 (gender)

## Results

### Epidemiology

The number of patients with ACS increased during the six study years. In 2004, there were 217,297 patients with a primary or secondary discharge diagnosis of ACS discharged from hospitals in South Korea. Increasing annually, the number of patients with ACS was 309,384 in 2009. Among the ACS patients, approximately 60% were male with similar proportions of males in each year from 2004 to 2009. The prevalence of ACS increased with age and, in 2009, approximately 64.1% of the ACS patients were 60 years of age or older. In 2004, the number of patients with MI was 119,775, representing 55.1% of the patients with ACS. The number of MI patients increased between the years 2004 and 2007 and then decreased until 2009. The number of MI patients was 148,989 in 2009, lower than the number of UA patients. The prevalence of ACS was 6.4 persons per 1,000 population members in 2009, an increase of 42.4% from the ACS prevalence in 2004 of 4.5 persons per 1,000 population members (Table 1).

**Table 1:** Epidemiology of ACS in South Korea from 2004 to 2009

	**2004**	**2005**	**2006**	**2007**	**2008**	**2009**
Prevalence						
Number of patients	217,297	238,517	257,889	283,829	291,088	309,384
Prevalence per 1000 population	4.5	5.0	5.3	5.9	6.0	6.4
Gender (%)						
Male	130,004	143,556	157,692	173,582	179,530	192,317
% of male	59.8%	60.2%	61.1%	61.2%	61.7%	62.2%
Age						
Over 60	128,528	143,745	155,569	174,423	183,408	198,212
% of over 60	59.1%	60.3%	60.3%	61.5%	63.0%	64.1%
Diagnosis						
UA	97,522	108,926	121,625	140,008	148,491	160,395
MI	119,775	129,591	136,264	143,821	142,597	148,989
Mortality						
Number of death	9,863	9,935	11,236	10,659	9,929	9,831
Mortality per 100 000 population	20.5	20.6	23.3	22.0	20.4	20.2
Diagnosis						
UA	227	227	240	280	225	252
MI	9,636	9,708	10,996	10,379	9,704	9,579

In 2004, the number ACS patient deaths was 9,863. That number increased annually and was 11,236 persons in 2006. However, since 2006, the number of ACS-related deaths has decreased: 10,659 people in 2007, 9,929 in 2008, and 9,831 in 2009. The mortality rate associated with ACS was 20.5 persons per 100,000 population members in 2004. That rate increased from 2004 to 2006 and then decreased until 2009. Deaths related to UA represented 2.3% of the ACS deaths while death due to MI represented 97.7% of all ACS deaths in 2009 (Table 1).

### Medical utilization

The total number of hospital visits due to ACS increased every year from 494,226 visits in 2004 to 786,827 visits in 2009. The hospital visits for outpatients increased sharply compared to those for inpatients and the outpatient-to-inpatient ratio was 9.7:1 in 2009 compared to 6.9:1 in 2004. The number of ACS-related inpatient days increased from 449,660 days in 2004 to 487,323 days in 2009. The number of inpatient days per visit decreased gradually from 7.18 days in 2004 to 6.62 days in 2009 (Table 2).

**Table 2:** Medical utilization due to ACS from 2004 to 2009

	**2004**	**2005**	**2006**	**2007**	**2008**	**2009**
Total hospital visits	494,226	543,033	597,739	695,024	733,313	786,827
Outpatient	431,602	477,581	530,747	663,056	663,056	713,139
Inpatient	62,619	65,444	66,971	73,124	70,232	73,654
Outpatient/Inpatient	6.9	7.3	7.9	9.1	9.4	9.7
Total inpatient days	449,660	457,618	452,187	510,564	484,354	487,323
Inpatient days per case	7.18	6.99	6.75	6.98	6.90	6.62

### Societal cost

The ACS-related NHI expenditures increased by 30.2% over the six study years from USD 208.7 million in 2004 to USD 334.3 million in 2009. The NHI payments for male ACS patients increased from USD 134.3 million in 2004 to USD 220.9 million in 2009 and were higher than those for females. The highest NHI expenditure by age group was USD 133.0 million, which occurred in 2009 for patients aged over 60 years. Based on treatment type, NHI hospitalization expenditures ranged between 83.4% and 84.1% of the total NHI costs, a percentage range that was approximately five times higher than that for outpatient care expenditures. Based on disease group, NHI payments associated with MI were USD 132.0 million in 2004 and USD 208.1 million in 2009, representing between 60.7% and 63.3% of the total NHI expenditures. The largest NHI expenditure proportion among the various medical institution types was 50.0%, paid to tertiary hospitals. By payment categories, the proportion expended for procedures and operations ranged between 33.6% and 34.0%, which was the highest level, followed by 27.1% to 27.4% expended for radiography, 11.4% to 12.1% expended for pharmaceuticals, and 10.5% to 10.7% for examinations (Table 3).

**Table 3:** NHI payments by groups from 2004 to 2009

	**2004**		**2005**		**2006**		**2007**		**2008**		**2009**	
**NHI payments ($ million)**	**208.7**	**(100.0%)**	**228.9**	**(100.0%)**	**280.4**	**(100.0%)**	**329.8**	**(100.0%)**	**312.1**	**(100.0%)**	**334.3**	**(100.0%)**
Gender												
Male	134.3	(64.4%)	146.2	(63.8%)	182.5	(65.1%)	215.1	(65.2%)	204.0	(65.4%)	220.9	(66.1%)
Female	74.4	(35.6%)	82.8	(36.2%)	97.9	(34.9%)	114.7	(34.8%)	108.1	(34.6%)	113.5	(33.9%)
Age												
<60	141.2	(36.3%)	80.2	(35.0%)	99.2	(35.4%)	114.4	(34.7%)	106.0	(33.9%)	112.4	(33.6%)
≥60	133.0	(63.7%)	148.8	(65.0%)	181.1	(64.6%)	215.4	(65.3%)	206.1	(66.1%)	221.9	(66.4%)
Type												
Inpatient	175.5	(84.1%)	191.0	(83.4%)	235.8	(84.1%)	278.7	(84.5%)	261.8	(83.9%)	281.3	(84.1%)
Outpatient	33.2	(15.9%)	38.0	(16.6%)	44.5	(15.9%)	51.1	(15.5%)	50.3	(16.1%)	53.0	(15.9%)
Diagnosis												
Myocardial infarction	132.0	(63.3%)	141.2	(61.7%)	171.2	(61.0%)	200.1	(60.7%)	193.8	(62.1%)	208.1	(62.2%)
Unstable angina	76.7	(36.7%)	87.8	(38.3%)	109.2	(39.0%)	129.7	(39.3%)	118.2	(37.9%)	126.2	(37.8%)
Medical institutions												
Tertiary hospitals	122.6	(58.7%)	129.6	(56.6%)	149.1	(53.1%)	168.2	(51.0%)	156.2	(50.0%)	173.7	(52.0%)
General hospitals	65.0	(31.1%)	74.7	(32.6%)	102.0	(36.4%)	126.9	(38.5%)	122.0	(39.1%)	124.7	(37.3%)
Hospitals	2.1	(1.0%)	2.6	(1.1%)	3.3	(1.2%)	4.9	(1.5%)	5.3	(1.7%)	5.7	(1.7%)
Clinics	0.7	(0.3%)	0.8	(0.4%)	0.9	(0.3%)	1.2	(0.4%)	1.3	(0.4%)	1.4	(0.4%)
Pharmacy	18.4	(8.9%)	21.2	(9.3%)	25.1	(9.0%)	28.5	(8.6%)	27.3	(8.8%)	28.7	(8.6%)
Categories												
Procedure and operation	70.6	(33.8%)	76.8	(33.6%)	94.9	(33.9%)	112.1	(34.0%)	105.3	(33.8%)	113.2	(33.9%)
Radiography	56.9	(27.3%)	62.0	(27.1%)	76.4	(27.3%)	90.2	(27.4%)	85.0	(27.3%)	91.3	(27.3%)
Pharmaceutical	24.3	(11.6%)	27.7	(12.1%)	33.1	(11.8%)	37.9	(11.5%)	36.2	(11.6%)	38.3	(11.4%)
Examination	22.1	(10.6%)	24.3	(10.6%)	29.5	(10.5%)	34.8	(10.5%)	33.3	(10.7%)	35.7	(10.7%)
Hospitalization	17.2	(8.2%)	18.7	(8.2%)	23.1	(8.2%)	27.3	(8.3%)	25.7	(8.2%)	27.6	(8.2%)
Others	17.6	(8.5%)	19.3	(8.4%)	23.4	(8.3%)	27.5	(8.3%)	26.6	(8.4%)	28.3	(8.5%)

The total SCs of ACS increased by USD 228.3 million over the six study years, from USD 659.9 million in 2004 to USD 918.2 million in 2009. Within the SCs associated with ACS, the proportion for indirect costs ranged from 50.5% to 58.8% of the total NHI costs, which was higher than the proportion for direct costs of between 41.2% and 49.5% (Table 4).

**Table 4:** Societal costs of ACS from 2004 to 2009

	**2004**		**2005**		**2006**		**2007**		**2008**		**2009**	
**Societal costs ($ million)**	**659.9**	**(100.0%)**	**718.1**	**(100.0%)**	**840.5**	**(100.0%)**	**880.6**	**(100.0%)**	**864.8**	**(100.0%)**	**918.2**	**(100.0%)**
Direct costs	272.4	(41.2%)	299	(41.6%)	364.8	(43.4%)	435.7	(49.5%)	402.8	(46.6%)	436.7	(47.6%)
Medical costs	265.5	(40.2%)	291.2	(40.5%)	356.3	(42.3%)	425	(48.3%)	392.1	(45.4%)	425.3	(46.3%)
Inpatient	227.6	(34.5%)	248	(34.5%)	305.8	(36.3%)	366.6	(41.6%)	334.8	(38.7%)	357.5	(38.9%)
Outpatient	37.9	(5.7%)	43.2	(6.0%)	50.5	(6.0%)	58.4	(6.7%)	57.3	(6.7%)	67.8	(7.4%)
Non-medical costs	6.9	(1.0%)	7.8	(1.1%)	8.5	(1.1%)	10.6	(1.2%)	10.7	(1.2%)	11.4	(1.3%)
Transportation	4.2	(0.6%)	4.9	(0.7%)	5.5	(0.7%)	7.1	(0.8%)	7.2	(0.8%)	7.9	(0.9%)
Caregiving	2.7	(0.4%)	2.9	(0.4%)	3	(0.4%)	3.5	(0.4%)	3.4	(0.4%)	3.5	(0.4%)
Indirect costs	387.5	(58.8%)	419.1	(58.4%)	475.7	(56.6%)	444.9	(50.5%)	462	(53.4%)	481.5	(52.4%)
Lost wage due to morbidity	10.3	(1.6%)	11.9	(1.7%)	13.9	(1.7%)	16.7	(1.9%)	16.9	(2.0%)	16	(1.7%)
Lost future income	377.2	(57.2%)	407.3	(56.7%)	461.8	(54.9%)	428.2	(48.6%)	445.1	(51.4%)	465.6	(50.7%)

The direct costs increased annually, except for 2008, from USD 272.4 million in 2004 to USD 436.7 million in 2009. Within the direct costs associated with ACS, the medical costs were estimated at USD 265.5 million in 2004, which was 40.2% of the 2004 total direct costs. Medical cost increased annually and was USD 425.3 million (46.3% of the total direct cost) in 2009. In addition, the direct MC for hospitalization was USD 227.6 million in 2004 and USD 357.5 million in 2009, approximately six times that for outpatient MC. The 2004 non-medical cost was approximately USD 6.9 million or 1.0% of the total 2004 expenditures. The NMC increased annually and was USD 11.4 million (1.3% of the total direct cost) in 2009. Of the direct NMC associated with ACS, transportation cost was USD 4.2 million in 2004, which increased yearly to USD 7.9 million in 2009. Caregiving cost was USD 2.7 million in 2004 and increased to USD 3.5 million in 2009 (Table 4).

The indirect cost in 2004 was USD 387.5 million, 58.8% of the total societal cost increasing to USD 481.5 million (52.4% of the total societal cost) in 2009. The indirect cost included lost wages of USD 10.3 million in 2004 and USD 16.0 million in 2009 and future lost income of USD 377.2 million in 2004 and USD 465.5 million in 2009 (Table 4).

## Discussion

Our analysis was based on 2004–2009 NHI medical claim data for the entire population in Korea. This data source was used to examine ACS-related healthcare utilization and associated expenditures in a real-world setting over a period of years.

The results of this study indicate that the societal costs associated with ACS are substantial. In Korea in 2005, the COI of circulatory diseases, including ACS, was 13.1% of the total COI with circulatory diseases being the second largest proportion of COI after that associated with neoplasms [1]. At the GDP level, the COI of circulatory diseases in Korea is reported to be comparable to that in other countries [16-22]. In this study, the indirect cost of ACS was approximately 55% of the total NHI cost. This result is attributed to the age distribution of the study population with the results showing high morbidity and mortality in those aged below 70 years, which is consistent with the results of other studies [4,23]. However, the high percentage of indirect costs in our study contrasts with the results of Johnston et al. [24]. The low proportion of indirect costs in the study by Johnston et al. may be due to differences in the measurement methods in the two studies; thus, it is difficult to compare the two results.

Several studies have shown that a MI diagnosis is associated with higher NHI expenditures than those associated with a diagnosis of UA [17,21]; results that are supported by our findings. Additionally, our study shows that NHI expenditures for procedures and operations including PCI and CABG represent the highest proportion of the total NHI cost, which supports the results of other studies on ACS-associated costs [17,18,20,21,25]. Since several studies have suggested that interventional approaches (such as PCI, stent, and CABG) reduce ACS-related morbidity and mortality [26,27], NHI expenditures on such approaches are likely to increase consistently. In this study, the NHI hospitalization cost was six times that of the NHI outpatient care cost. Moreover, NHI expenditures in tertiary hospitals accounted for more than 50% of the total NHI payments. Collectively, these findings indicate that ACS is a societally important condition, one that requires appropriate treatment.

Over the study period, the number of patients with ACS increased continuously from 217,297 in 2004 to 309,384 in 2009. Annually over that period, between 130,004 and 192,317 (approximately 60%) were men. As with many other diseases, the prevalence of ACS increases with age. In our study, approximately 50% of the ACS patients were over the age of 60 years. The proportion of ACS patients with UA was 44.9% in 2004, which is lower than that of MI patients (55.1%). Over the study period, the number of patients with UA increased more sharply than that of MI patients and there were more UA patients than MI patients in 2009.

20.2 and23.3 persons per 100,000 population members, representing approximately 4% of the total deaths and 17% of the cardiovascular disease deaths in Korea [1]. While the prevalence of ACS increased over the study period, the mortality rate decreased. This result can be attributed to improvements in the early detection and treatment of ACS. Although the elderly (those aged over 70 years) had a high morbidity in this study, the mortality rate in patients at a working age was also high. This trend can lead to increased indirect costs, especially those associated with future income. Of the patients in this study who died due to ACS, MI was associated with 98% of the deaths. This is likely attributed to disease progression of UA, which proceeds to MI with deterioration. We found no other studies that showed a relationship between ACS prevalence and subsequent mortality.

Over the study period, ACS-related hospital visits increased for both outpatients and inpatients. Since the outpatient visits increased more rapidly than that of the inpatients, the outpatient-to-inpatient ratio increased annually. Recent studies have shown that same-day discharge after procedures such as PCI and coronary angiography is feasible and safe [28-30]. Moreover, because of factors such as patient satisfaction, medical costs, and waiting lists the prevalence of same-day discharge is increasing. Accordingly, that trend is likely to have affected the outpatient-to-inpatient ratio in this study. While the outpatient visits continued to increase during our study, inpatient days decreased consistently from 7.18 days in 2004 to 6.62 days in 2009. This decrease is less than that reported by others [16,18], except for a study conducted in the United States [16,18,20,25]. Country specific differences in inpatient day results have been attributed to the country’s type of healthcare system.

### Limitation

This study has several limitations related to measuring costs. First, we included only the cost of caregiving that was provided by paid caregivers. To calculate caregiving costs more accurately, the cost of the informal care provided by relatives should also be included. To that end, information on relatives’ characteristics such as gender, age, and occupation is required. Because of insufficient information on the characteristics of the caregivers we were unable to include costs related to informal care. Second, we were unable to estimate some items because of data restrictions and objective quantification difficulties. For example, the cost for emergency services and the intangible costs such as those related to pain and emotional anxiety due to ACS were excluded from the study. Third, notwithstanding uncertainties about the data, no sensitivity analysis to test the robustness of our results was performed. If such limitations are addressed in a future study, more accurate measurements of SC of ACS could be achieved.

## Conclusion

This 2004 to 2009 study showed that the number of patients with ACS in Korea has sharply increased and that the societal cost of ACS in Korea is markedly high. ACS is likely to remain a leading cause of hospitalization, due to the aging Korean population and because of an increase in risk factors for coronary heart disease. It is expected that the proportion of the Korean population that is aged 65 years and older, and therefore at a high risk of ACS, will increase until 2030. Thus ACS will continue to be a major healthcare disease in the near future. This, coupled with the continued presence of high risk factors associated with ACS (such as obesity and diabetes) suggests that early and effective management of ACS is required to reduce ACS-related mortality and morbidity. Hence, significant public and private healthcare resources will continue to be required to prevent and treat ACS. The findings of this study suggest that further research should be undertaken to discover ways to reduce the economic effects of ACS on the Korean population.

## Competing interests

The authors declare that they have no competing interest.

## Authors’ contributions

All authors read and approved the final manuscript.

## Pre-publication history

The pre-publication history for this paper can be accessed here:

http://www.biomedcentral.com/1471-2261/13/55/prepub
